# An easy maneuver to screen for moderate-to-severe obstructive sleep spnea: the Simmons Chin Press and Tongue Curl

**DOI:** 10.5935/1984-0063.20200092

**Published:** 2021

**Authors:** Gerard Joseph Meskill, Kelly Kincheloe, Jerald Howard Simmons, Sarah Dennis Meskill

**Affiliations:** 1 Tricoastal Narcolepsy and Sleep Disorders Center, Sleep Medicine - Pearland - Texas - United States.; 2 Comprehensive Sleep Medicine Associates, Sleep Medicine - Houston - Texas - United States.; 3 Baylor College of Medicine, Pediatric Emergency Medicine - Houston - Texas - United States.

**Keywords:** Sleep Apnea, Obstructive, Physical Examination, Diagnostic Techniques, Respiratory System, Otological, Neurological

## Abstract

**Objectives:**

To demonstrate that the Simmons chin press and tongue curl (SCPTC) correlates with diagnosis of moderate-to-severe obstructive sleep apnea (msOSA) by itself as well as irrespective of the presence of other associated features.

**Material and Methods:**

A consecutive sample of 1,911 sleep studies performed on adult patients from 2/8/2017 to 3/5/2019 was analyzed. The SCPTC exam maneuver was performed on each patient, followed by home sleep apnea testing or in-laboratory polysomnography. The AASM hypopnea 1B 4% desaturation criteria were utilized for scoring to correlate results to existing literature on morbidity and mortality. A chi-squared using low and high SCPTC score was performed for the outcome of msOSA. Known significant predictors of OSA were dichotomized for comparison and a multiple logistic regression was performed.

**Results:**

1,708 patients qualified for inclusion: 902 males (52.8%) and 806 females (47.2%) with a mean age of 49.4 and a mean body mass index (BMI) of 28.6. A high SCPTC score correlated with an odds ratio (OR) of 2.49 (95% CI: 2.03-3.04, p<0.001) for msOSA. A multiple logistic regression analysis including other risk factors for msOSA demonstrated that high SCPTC scores had an odds ratio for msOSA of 1.77 (95% CI: 1.40-2.23; p<0.001).

**Conclusion:**

The SCPTC is a reproducible physical exam feature that can be utilized by healthcare providers to screen for patients with msOSA.

## INTRODUCTION

Untreated obstructive sleep apnea (OSA) can lead to hypertension, coronary artery disease, heart failure, stroke, and insulin resistance and type 2 diabetes^1^. Those experiencing intermittent hypoxemia have an increased all-cause mortality risk^1^. Current prevalence studies estimate that OSA affects 34% of men and 17% of women in the US^2^, while moderate-to-severe OSA (msOSA) affects 13% of men and 6% of women^3^.

Since most patients with sleep disorders are evaluated first by their primary care providers (PCPs), the American Academy of Sleep Medicine (AASM) recommends that PCPs screen all high-risk patients, even if asymptomatic^4^. High-risk criteria often are focused on metabolic and cardiovascular (CV) disorders^5^. Other screening tools to identify those at risk rely on symptoms of sleepiness (e.g., Epworth sleepiness scale [ESS]) or characteristic features, such as obesity or snoring volume (e.g., STOP-BANG and Berlin questionnaires).

If OSA were limited to individuals who were overweight, sleepy, or with loud snoring, the aforementioned screening tools might be sufficient. However, these tools underserve patients with atypical features, and studies have demonstrated they are unreliable in predicting OSA in asymptomatic adults^6^. In fact, the majority of OSA disease side effects rest with patients who are asymptomatic since sleepiness (ESS>10) occurs in only 28% of moderate and 35% of severe OSA cases.^7^. Further, significant OSA burden exists in individuals with normal body habitus^8,9^. This becomes critically important when considering the risk of cardiovascular and cerebrovascular morbidity and mortality rests with individuals with OSA under 70 years old, irrespective of symptoms^10^. In females aged 20-70 years old, there is no relationship between OSA and daytime sleepiness^11^. Snoring frequency actually decreases after age 60, even as OSA prevalence continues to rise^12^. While snoring frequency and severity, excessive daytime sleepiness, male gender, and obesity are the dogma of OSA screening in the community, these observational tools unfortunately leave a significant proportion of the population undiagnosed. US medical training affords little to no formal sleep education^13^, resulting in PCPs being unfamiliar with the effects and long-term ramifications of sleep disorders^14^. These may explain why 80 percent of msOSA cases are thought to be undiagnosed^15^. The financial burden associated with untreated OSA in the US is estimated to be $148.9 billion annually secondary to comorbidities and mental health disorders, motor vehicle collisions, workplace accidents, and lost productivity^6,16^.

The Simmons chin press and tongue curl (SCPTC) is a physical exam maneuver performed at the bedside that mimics the position of the mandible and tongue during sleep to assess dynamic effects on upper airway patency during sleep. It is a simple, fast, easily interpreted maneuver that can identify physiologic causes of airway obstruction, regardless of whether they are related to body habitus, excess pharyngeal soft tissue, and/or craniofacial architecture. We aim to demonstrate that it has the ability to identify individuals at risk of msOSA.

## MATERIAL AND METHODS

This is a retrospective cross-sectional study of consecutive adult patients who presented to a sleep practice in an urban setting from 2/8/2017 to 3/5/2019. Patients were included if they had the SCPTC performed and underwent a diagnostic sleep study. Sleep studies using an oral appliance or for post-surgical assessment were excluded. In agreement with AASM hypopnea 1B criteria, home sleep apnea tests tabulating 3% rather than 4% desaturation hypopneas were excluded. The hypopnea 1B criteria were selected to correlate the results to existing literature on cardiovascular morbidity risk, which utilized these same criteria. The SCPTC was performed by 1 of 6 practitioners. Each clinician watched a video on how to perform the maneuver.

The SCPTC starts with the patient lying supine with the mouth closed, teeth together, and the temporomandibular joint (TMJ) in an occlusive relation. First, baseline nasal breathing is established. Next, the examiner places gentle pressure on the chin to guide the TMJ into the most retruded centric relation (“chin press”). The patient again is instructed to breathe nasally as the examiner observes for obstruction. The patient then is instructed to touch the tip of the tongue to the hard palate and slide it as posteriorly as possible (“tongue curl”) while the examiner repeats the chin press and observes for obstruction. Each maneuver is scored separately: no obstruction scores 0, partial obstruction 1, and complete obstruction 2. Partial obstruction is defined as increased resistance to breathing as observed by the patient or the examiner. Complete obstruction is defined as a cessation of airflow despite effort, even if momentarily. The two scores are summed to produce the SCPTC score with 4 total points possible. The SCPTC scores were divided into low (0-2) and high (3-4) categories. Demographic characteristics (age and sex), anthropomorphic data (BMI and neck circumference), and a questionnaire on daytime sleepiness (ESS) were extracted from the medical record. Age was dichotomized to <50 years old or ≥50 years old, neck circumference to <17 inches or ≥17 inches, BMI to <35 or ≥35, and the ESS to <11 or ≥11. These cutoffs were chosen to match current screening assessments (i.e., age and BMI correlate to STOP-BANG questionnaire questions; neck circumference of ≥17 inches has been associated with increased risk of OSA^17^; ESS≥11 is the established threshold for EDS^18^). An apnea-hypopnea index (AHI) of ≥15 was considered reference standard for msOSA.

A chi-squared using low and high SCPTC score was performed for the outcome of msOSA. In cases with complete data, a multiple logistic regression including known significant predictors of OSA was performed. The multiple logistic model was evaluated for fit by using a receiver operating characteristic (ROC) curve. Data were analyzed using Stata software, version 14.1 (StataCorp). This study was approved by Texas Orthopedic Hospital IRB.

## RESULTS

**Figure 1:**
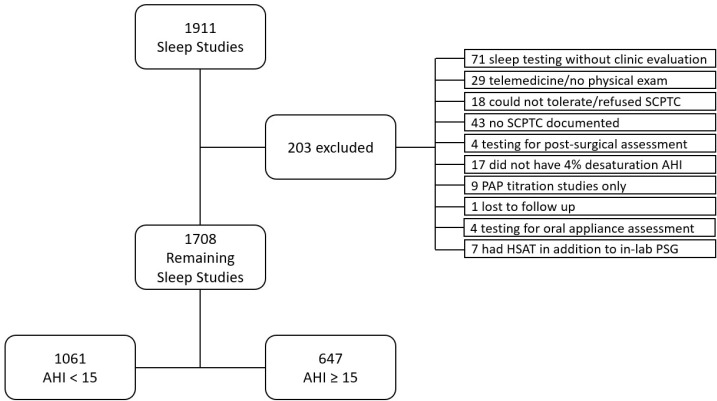
Flow chart of inclusion and exclusion criteria. **Notes:** SCPTC = Simmons chin press and tongue curl; AHI = Apnea hypopnea index; PAP = Positive airway pressure; HSAT = Home sleep apnea test; PSG = Polysomnogram.

There were 1,911 consecutive sleep studies. 203 sleep studies were excluded (see Figure 1). The remaining 1,708 patient charts were analyzed (Table 1). There were 71 patients (4.1%) who did not have a neck circumference documented and 13 (0.7%) who did not have an initial ESS documented. In the chi-squared analysis, a high CPTC score had an odds ratio for msOSA of 2.49 (95% CI: 2.03-3.04, *p*<0.001). The multiple logistic regression analysis including other risk factors for msOSA demonstrated that a high SCPTC score had an odds ratio of 1.77 (95% CI: 1.40-2.23, p<0.001) for msOSA (Table 2).

**Table 1:** Demographic information on patient population.

	AHI < 15 mean (range) n=1061	AHI ≥ 15 mean (range) n=647	Total (percent)/mean	p-value
Male	462	440	902 (52.8%)	<0.05
Age (years)	46.1 (18-88)	54.9 (21-90)	49.4	<0.05
BMI (kg/m2)	28.6 (15.1-54.1)	34.5 (17-82.2)	30.8	<0.05
NC (inches)	15 (10-21.5)	16.9 (11.5-24.5)	15.7	<0.05
ESS	11.3 (0-24)	11.1 (0-24)	11.2	0.49
“High” SCPTC score 3-4	228	419	647 (37.9%)	<0.05

**Notes:** BMI = Body mass index; NC = Neck circumference; ESS = Epworth sleepiness scale; SCPTC = Simmons chin press and tongue curl.

**Table 2:** Logistic regression for moderate-to-severe OSA.

	OR	95% confidence	p-value
SCPTC score	1.77	1.40-2.23	<0.001
ESS ≥11	1.11	0.88-1.40	0.38
Male sex	1.87	1.43-2.46	<0.001
Age ≥50 years old	2.81	2.22-3.56	<0.001
NC ≥17 inches	2.52	1.90-3.33	<0.001
Body mass index ≥35kg/m^2^	3.31	2.49-4.41	<0.001

**Notes:** SCPTC = Simmons chin press and tongue curl; ESS = Epworth sleepiness scale; NC = Neck circumference.

## DISCUSSION

Undiagnosed OSA is an enormous burden on the US healthcare system, and current screening tools have low sensitivity toward identifying msOSA in patients without metabolic or CV high-risk factors. Individuals with high SCPTC scores have a significant increased risk of msOSA irrespective of the presence of traditional risk factors, such as sleepiness, older age, male sex, obese body habitus, and increased neck circumference. These other features (except sleepiness) all have predictive value in identifying msOSA (see Table 1), but they omit a substantial patient population, and therefore are insufficient for screening.

We suspect the SCPTC’s diagnostic power is attributed to the maneuver’s reproduction of the retro-position of the tongue and jaw during sleep, thus simulating OSA pathophysiology. The tongue and TMJ muscles exhibit decreased tone during sleep, leading to retro-position of these structures and narrowing of the upper airway. Established exam features (e.g., palate score) rely on static anatomy and do not account for the interrelation between craniofacial anatomy and physiologic changes in musculoskeletal positioning during sleep.

Recently, clinicians have taken interest in analyzing the benefit of treating asymptomatic OSA. One study concluded that treating patients with msOSA did not prevent secondary CV events, but the subjects in the study had inadequate treatment due to poor compliance (average of 3.3 hours per night), and thus the conclusion is not justified^19,20^. Conversely, Peker et al. (2016)^21^ demonstrated that compliant continuous positive airway pressure (CPAP) use significantly reduced secondary CV events in non-sleepy OSA patients, while non-compliant use did not. Fu et al. (2017)^22^ demonstrated that CPAP use reduces all-cause mortality in patients with severe OSA.

The SCPTC has the potential to markedly improve detection of OSA. Based upon these results, we recommend any patient with a high SCPTC score to have an evaluation via HSAT or in-laboratory PSG, as the score itself is not sufficient to diagnose msOSA. Sleep disorders screening is limited or absent in most fields of medicine, and therefore dissemination of this technique could have substantial ramifications for secondary disease prevention with subsequent healthcare cost reduction.

## CONCLUSION

This study demonstrates that the SCPTC is a simple physical exam feature that can be utilized by healthcare providers to screen for patients with msOSA.

### Limitations

First, this was a retrospective cross-sectional study with some missing data, though the missing data was minimal and did not affect the overall ability to interpret the regression. Second, this is not a randomized controlled trial, but given this is a screening maneuver that design is not appropriate. Third, the patients analyzed presented due to concerns about a sleep disorder, biasing the study toward those with sleep pathology. Fourth, race and ethnicity were not documented, and therefore we cannot comment on their role on OSA risk.
